# What will make a difference? Assessing the impact of policy and non-policy scenarios on estimations of the future GP workforce

**DOI:** 10.1186/s12960-017-0216-1

**Published:** 2017-06-28

**Authors:** Caroline O. M. Laurence, Jonathan Karnon

**Affiliations:** School of Public Health, The University of Adelaide, North Terrace, Adelaide, Australia

**Keywords:** General practice, Workforce, Simulation modelling, Cost

## Abstract

**Background:**

Health workforce planning is based on estimates of future needs for and supply of health care services. Given the pipeline time lag for the training of health professionals, inappropriate workforce planning or policies can lead to extended periods of over- or under-supply of health care providers. Often these policy interventions focus on one determinant of supply and do not incorporate other determinants such as changes in population health which impact the need for services.

The aim of this study is to examine the effect of the implementation of various workforce policies on the estimated future requirements of the GP workforce, using South Australia as a case study. This is examined in terms of the impact on the workforce gap (excess or shortage), the cost of these workforce policies, and their role in addressing potential non-policy-related future scenarios.

**Methods:**

An integrated simulation model for the general practice workforce in South Australia was developed, which determines the supply and level of services required based on the health of the population over a projection period 2013–2033. The published model is used to assess the effects of various policy and workforce scenarios. For each policy scenario, associated costs were estimated and compared to baseline costs with a 5% discount rate applied.

**Results:**

The baseline scenario estimated an excess supply of GPs of 236 full-time equivalent (FTE) in 2013 but this surplus decreased to 28 FTE by 2033. The estimates based on single policy scenarios of role substitution and increased training positions continue the surplus, while a scenario that reduces the number of international medical graduates (IMGs) recruited estimated a move from surplus to shortage by 2033. The best-case outcome where the workforce achieves balance by 2023 and remains balanced to 2033, arose when GP participation rates (a non-policy scenario) were combined with the policy levers of increased GP training positions and reduced IMG recruitment. The cost of each policy varied, with increased role substitution and reduced IMG recruitment resulting in savings (AUD$752,946,586 and AUD$3,783,291 respectively) when compared to baseline costs. Increasing GP training costs over the projection period would cost the government an additional AUD$12,719,798.

**Conclusions:**

Over the next 20 years, South Australia’s GP workforce is predicted to remain fairly balanced. However, exogenous changes, such as increased demand for GP services may require policy intervention to address associated workforce shortfalls. The workforce model presented in this paper should be updated at regular intervals to inform the need for policy intervention.

**Electronic supplementary material:**

The online version of this article (doi:10.1186/s12960-017-0216-1) contains supplementary material, which is available to authorized users.

## Background

### Workforce challenges in Australia

As in many other countries, Australia is facing a number of health challenges that put pressure on the supply of medical practitioners [1, 2]. These include an increasing demand for health services arising from an ageing population, increasing chronic illness, greater complexity of care and consumer expectations; changing demographics within the medical workforce with more doctors working part-time and an ageing workforce; and changing models of care which require a different mix of skills; and new technologies. In addition to these, the distribution of the medical workforce in Australia has been a persistent problem, with shortages in rural and remote as well as outer metropolitan areas [3, 4].

In response to these challenges, the Australian government has implemented a number of policies and strategies with the majority directed at geographic maldistribution. These have either focused on improving recruitment through education and training, financial incentives and recruitment of international medical graduates or retention through locum support, retention payments and training [2]. In addition to these, a key policy has been to increase the number of graduates by expanding the number of medical schools in Australia, many of which are regionally located, with the aim of increasing the overall supply of medical practitioners, as well as those working in rural areas [5].

Some of these policies have been effective. Australia now has one of the highest rates of medical graduate completions in the world (15.3 graduates per 100,000), ranking third of 29 OECD countries [6]. It also ranks in the top 10 of OECD countries in the number of doctors per capita, with 3.5 doctors per 1000 population [7]. Despite this, the growth is uneven, with greater growth of specialists compared to GPs and disparities in geographic location of the workforce [5].

In order to respond to the workforce challenges, governments need to implement policies that meet the desired results, an overall balanced workforce, and for Australia, an evenly distributed workforce in relation to population need.

The role of health workforce planning is to inform decision-making to ensure we have the right skills in the right place at the right time to provide the right services to the right people. Given the pipeline time lag for the training of health professionals, inappropriate workforce planning or policies can lead to extended periods of over- or under-supply of health care providers [8]. An important part of this planning process is estimating the future needs for, and supply of health care services and simulation models are often used for this purpose [9, 10]. These simulation models allow governments to test the effect of different policies of workforce supply over a projection period through scenario analysis. However, the approach or methods taken in these models vary and have tended to simplistic or supply focussed.

### Australian health care system

Workforce planning is also influenced by the health care system in which it occurs. Its structure and funding mechanisms determine the health workforce size and mix but also what policies and strategies are possible to address workforce problems.

The Australian healthcare system involves multiple funders and health care providers, with responsibilities split across different levels of government but also between the government and private or non-government sectors [11]. The funding of health care is primarily the responsibility of the Australian Commonwealth Government through health insurance and payments to the States and Territories, while the State and Territories are primarily responsible for the direct provision of services.

Universal access to health services is provided through the publicly funded system, Medicare. It provides free or subsidised access to medical services in hospital and primary care settings and pharmaceuticals [11]. The majority of health expenditure is spent on hospitals (40.8%) and primary care (38.2%) [11]. The state and territory governments are primarily responsible for public hospital services, including outpatient services, while the private sector provides health care through private practitioners, private hospitals and pathology services.

Most often, the first point of contact with the health systems for Australians occurs in primary care mainly through general practitioners (GPs). Most GPs are self-employed and they act as gate keepers to other levels of the health systems, referring patients to specialists as well as diagnostic and allied health services. GPs are the largest medical speciality group in Australia comprising 33.1% of the medical workforce in 2015 [12].

As such, combined with primary care expenditure, medical workforce strategies in Australia have largely focused on GPs as a surplus or shortage in this discipline has the greatest impact on the health of the population, the provision of health care and expenditure [13].

### Health workforce planning in Australia

Over the last 30 years, health workforce planning in Australia has gone through a number of approaches and differing estimations in the medical and GP workforce. During this time, estimations have fluctuated from GP surpluses to shortages, with various policies implemented to address these. In the 1990s, with the establishment of the Australian Medical Workforce Committee (AMWAC), benchmarking was the dominate approach, with estimations of an over-supply of 2419 FTE GPs, particularly in urban areas [14]. In 2000, AMWAC estimated the supply and requirements for GPs from 1999 to 2010 and concluded that the number of GPs would increase over this period, but that distribution would be uneven with lower GP to population ratios in rural areas [15]. Further, planning estimations undertaken by AMWAC in 2005, estimated a shortfall of 639 GPs by 2013 and determined that balance could be achieved if the number of GP graduates increased from 2007 [16]. The key policies that resulted from these planning exercises were focused on medical schools and in migration of doctors. In the 1990s, there were capping of medical places and limiting the number of overseas doctors entering the workforce. By the mid 2000s, the policy directions reversed, with an increase in medical places, enabling an increasing number of overseas trained doctors to enter the Australian workforce, and increasing GP training places to address the estimated shortages in this discipline [16]. In order to address the issue of maldistribution, government strategies to attract and retain GPs in rural and remote areas were also introduced that included bonded medical places, regional medical schools, overseas doctors working in designated areas of need, establishment of University Departments of Rural Health and a rural pathway in GP vocational training [17].

A number of researchers have also undertaken estimations of future GP supply. In 2006, Joyce et al [13] projected the future supply of the medical workforce in Australian and concluded that there was likely to be a shortage of GPs by 2012, despite an overall surplus in the medical workforce. Harrison et al. [18] estimated GP supply until 2020 taking into account utilisation patterns and population changes and estimated that between 6101 and 7481 additional GPs would be required to meet service requirements, particularly in rural and regional areas.

More recently, Health Workforce Australia (HWA) undertook an extensive workforce planning project known as Health Workforce 2025 [1]. Within this project they estimated national workforce projections up to 2025 for a number of disciplines including medicine, nursing and midwives [1, 19]. In their modelling, demand was measured by utilisation and a stock and flow approach was used to determine supply. While this project added to health workforce planning research, it did not accommodate the health needs of the population. Building on this, we developed a simulation model for the general practitioner workforce in Australia that moved beyond utilisation as a measure of demand to one that incorporated changes in population health need, the first time this has been done for this workforce in Australia [20]. This model was based on the approach developed in Canada by Birch and Tomblin [21, 22], and we were able to estimate the number of general practitioners (GPs) required in South Australia over the next 20 years (2013–2033) [20].

While both these models provide estimates of future supply requirements, they also provide a method for assessing the impact of a range of workforce planning scenarios. The scenarios can focus on reducing or increasing supply as well as incorporating the impact of changes in the population and health need that affect demand. For example, the HWA modelled the impact of changes in demand (high and low) and self-sufficiency (reduced reliance on overseas trained doctors) [19]. What is rarely considered in this planning process is the impact of the policies in terms of cost or the impact of exogenous changes to needs and demands. Therefore, the aim of this study is to examine the effect of various workforce policies on the estimated future GP workforce, using South Australia as a case study. This is examined in terms of their impact on the workforce gap (excess or shortage) under alternative potential non-policy-related scenarios and the cost of these workforce policies.

## Methods

**Fig. 1:**
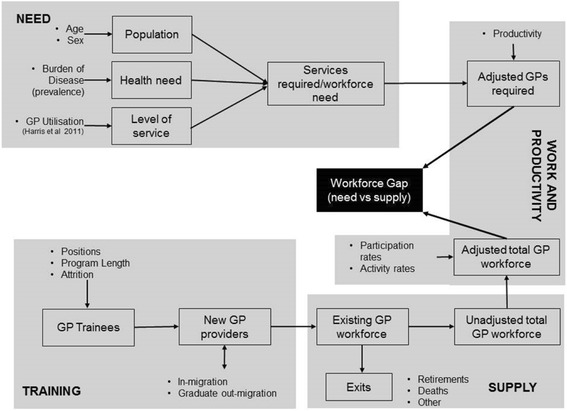
Overview of the planning model for GP

A simulation model was developed for the GP workforce in SA. Details of the model design have been published elsewhere [20] but an overview of the model is provided in Fig. 1 [20]. Briefly, the model has two sub-models—supply and needs. To estimate supply, a stock and flow approach was used which represented the flow of the current stock of GPs (by age groups and sex), and new entrants (graduates and in-migration) between states of employment that reflected location (urban and rural), work status (part-time and full-time), and exits from the workforce (permanent and temporary). Transition probabilities were estimated for the movement of GPs between the employment states, which were calibrated so that the model accurately predicted the observed distribution of GPs across the employment states between 2004 and 2013. To estimate the need for GP services, the age- and sex-specific prevalence and incidence of disease categories in SA were mapped to GP activity using data from the Bettering Evaluation and Care of Health (BEACH) Program to estimate service requirements (numbers of consultations) by disease, age and sex. The model tested three different scenarios based on assumptions regarding utilisation rates and the health of the population. In the first scenario, constant utilisation rates and incidence and prevalence rates were applied, based on rates estimated using data from 2003. In the second scenario, constant incidence and prevalence rates were applied but utilisation rates were assumed to have increased. An annual increase in utilisation per unit of need of 1.12% was applied to predict observed service use in 2013 [20]. In the third scenario, constant utilisation rates were applied to incidence and prevalence rates that were estimated to have increased annually by 2% between 2003 and 2013 (to predict observed service use in 2013). Scenario 2 was specified as the base-case scenario. The number of FTE GPs required to meet the predicted need for consultations were estimated annually to the year 2033.

### Baseline scenario

Beyond 2013, the baseline scenario represents changes in population demographics, as predicted by the Australian Bureau of Statistics [23], assuming constant age- and gender-specific disease incidence and prevalence rates to represent need and a continuing upward trend in level of services. The demographic changes for this scenario are depicted in graphs in Additional file 1. Known changes in GP training places up to 2015 were included and then assumed to be constant.

### Policy- and non-policy-based scenarios

The baseline scenario was adapted to assess the effect of various policy options, as well as uncertainty around the baseline predictions of workforce supply and population need. Four scenarios were applied to the needs sub-model and three to the supply sub-model. The scenarios selected represent major issues facing the provision of GP services in Australia based on a review of the literature, including analysis of data on GP workforce trends and input from stakeholders through a Reference Group. The Reference Group comprised of representatives and experts from key GP and health organisations including the Royal Australian Collage of General Practitioners, the Australian College of Rural and Remote Medicine, SA Department of Health, Medicare Locals, the Rural Doctors Workforce Agency, General Practice Education and Training Ltd and Health Workforce Australia. The rationale and details for each scenario are summarised in Table 1. Relevant scenarios were also combined to explore their joint effect on the results.

**Table 1:** Summary of scenarios used in the model

Sub-models	Scenario	Description	Rationale/source	Measures	Policy or non-policy scenario^a^	Scenario impact in 2033^b^
Need and Supply	1. Base scenario	Uses base year data—2003/04 Projections commence 2013 No changes except annual increase in level of service and population changes	Various (see [20] for details)	Various (see [20] for details)		
Need	2. Reduced illness of the population	Changes in population incidence and prevalence of disease.	Vos et al [32] and Goss [33] predicted changes in incidence and prevalence for selected injuries and illnesses.	Changes in estimated prevalence/incidence levels from base year Three changes estimated for years 2013, 2023 and 2033	Non-policy	−3.35% less need
	3. Increased role substitution	Increased use of practice nurse in GP consultations.	Bettering Evaluation and Care of Health (BEACH) data on proportion of consultations/conditions managed where nurse involved [44]. In 2010–11, 9% of all encounters involved a practice nurse in patient care.	10% of all estimated GP consultations undertaken by practice nurses.	Policy	−10.00% less need
	4. Increased Prevention	Change in preventive consultations as proportion of all consultations	Over 50% of GP consultations are for the management of chronic diseases and this is increasing [45]. A number of key strategies support increased preventative activity such as screening, immunisation and health checks [46, 47].	A 5% increase in the proportion of prevention consultations	Policy	0.05% more need
	5. Increased visits to the GP	Change in average number and length of consultations.	Average number and length of consultations is an important measure of need. Changes in utilisation can result from the introduction of new Medicare items [48] or influenced by the attributes of the GPs [49].	5% increase in average length of consultations by age/sex and 2011–12 estimates of average number of consultations from FMRC customised analysis.	Non-policy	7.36% more need
Supply	6. Increased GP training places	Increase in number of GP training places.	GP training positions has a direct impact on workforce supply.	SA proportion of an increase in 500 training positions nationally between 2015 and 2033 based on historic rates.	Policy	5.04% more supply
	7. Reduced Participation	Change in proportion of GP stock working full or part-time.	AIHW Medical Labour Force Survey shows that between 1999 and 2009, the number of hours worked by GPs fell from 45.6 to 42.2 per week [50]. Working hours have declined more for male GPs (by 7.4%) than for female GPs (6.4%) [13]	A 25% increase in the number of urban full-time male GPs who move to part-time work.	Non-policy	−0.90% less supply
	8. Reduced IMG recruitment	Decrease in number of IMGs entering the workforce for rural SA	Government goal from 2004 is national self-sufficiency and less reliance on immigration as a workforce strategy [40].	Change in IMGs entering rural GP by age, sex and work status by 25% from 2014	Policy	−6.29% less supply

^a^Policy scenarios reflect factors that can be influenced by government policies, non-policy scenarios reflect factors that may change in the absence of a government policy. Some scenarios are defined as policy and non-policy as they can be targeted by policy, but also change without a policy intervention

^b^Impact is based on percentage difference in demand or supply of each scenario from the baseline scenario in 2033

In assessing the scenario outcomes, it was assumed that the policy objective was to achieve a balanced workforce over the projection period and the best-case scenario/s represent the outcome closest to achieving a balanced workforce and the worst-case scenario/s result in an increased gap between supply and demand. A balanced workforce was defined as a difference of less than ±5% between supply and demand (with demand as denominator), which is equivalent to maximum difference of ±112 FTE GPs in 2033.

### Cost of policy scenarios

For each policy scenario, we estimated the cost of the policy from a government perspective and compared it to the cost of the baseline scenario for that policy area. Details of the item costs and data sources for each policy and baseline scenario are shown in Table 2.

**Table 2:** Details of item costs and data sources for costing the policy scenarios

Policy	Item	Source	Costs
Increased role substitution (scenario 3)	Average cost of nurse consultations	Medicare Australia—unreferred attendances practice nurse items, 2013−2016 [51]	Average cost for nurse consultations was based on total services and total benefits averaged over 4 years. Average cost of nurse consultations: AUD $13.74
	Average cost of GP consultations	Medicare Australia—unreferred attendances VR, non−VR and enhanced primary care items, 2013–2016 [51]	Average cost for GP consultations was based on total services and total benefits averaged over 4 years Average cost of GP consultations: AUD $48.48
Increased GP training positions (scenario 6)	Cost of training a GP registrar	GPET Annual Report to June 2014 [24]	Annual cost based on 5-year average cost per FTE training week of AUD $962 × 52 weeks (2009–2013) Annual cost of one training position: AUD $50,024 Represents total expenditure by Regional Training Provides on the AGPT program including recurrent and non-recurrent costs
Reduced IMG recruitment (scenario 8)	Average cost to recruit an IMG	International Recruitment Strategy (IRS), Department of Health Budget Portfolio statements 2014–2015 [52] Rural Health Workforce Australia Annual reports 2013 to 2016 [53–56]	Four-year average target for IMGs recruited through the IRS Four-year average cost of IRS program Average cost of recruitment of IMG: AUD$28,769

For the policy based on increased GP training places, an annual cost for training a GP registrar was estimated using program expenditure data from the General Practice Education and Training Limited [24]. The 5-year average cost per FTE training week (2009–2013) of AUD$962 was then used to determine an annual cost of training for a FTE GP registrar of AUD$50,024. This was then applied to the number of places in the baseline and increased GP training places scenario to determine total costs over the period 2014–2033. It was assumed that no additional medical students would be enrolled, only additional training places for general practice and that a higher proportion of medical graduates would become GPs. The average length of training was 3 years.

For the increased role substitution policy, the cost of a nurse providing 10% of all GP consultations were compared to the baseline costs of a GP providing all the consultations. An average cost for nurse consultations was estimated using a 4-year average (2013–2016) of total benefits and services for unreferred attendances for Medical Benefit Schedule practice nurse items. The average cost per nurse consultation was AUD$13.74. The average cost for GP consultations was estimated in a similar way using a 4-year average of total benefits and services for unreferred attendances excluding practice nurse items. The average cost per GP consultation was AUD$48.48. These were then applied to the baseline scenario, where all services were provided by the GP. For the policy scenario, the net average cost of nurse consultations minus the average cost of GP consultations was applied to 10% of the estimated GP consultations for the period 2014 to 2033. We assumed that there were a sufficient number of nurses with the appropriate skills to take on the extra consultations and that GPs would not increase their service provision.

The cost for the reduced international medical graduate (IMG) scenario was done in two parts. First, a cost for the recruitment of an IMG was estimated and then the cost of training additional Australian graduates to fill the gaps resulting from a reduction in IMGs was calculated using the GP training places cost described above. The cost of recruiting an IMG was derived from the annual budget allocation to Rural Workforce Agencies for the International Recruitment Strategy (OTD recruitment strategy and OTD additional assistance scheme) from the Department of Health divided by the recruitment target for the Strategy. This provided an average cost over 4 years per IMG recruited of AUD$28,769.

An annual discount rate of 5% was applied to estimate the net present value of future costs based on Australian guidelines [25, 26]. The investment of the funds required for spending in the future is assumed to yield a real rate of return and so costs are discounted to allow for the opportunity costs of current spending relative to delayed spending.

### Statistical analysis

Probabilistic sensitivity analysis (PSA) was undertaken to represent the uncertainty around the expected need for and supply of GP services and the effects of the alternative policy options. For the base-case scenario, convergent sets of input parameter values were identified as those that predicted observed service use in 2013 with less than a 5% absolute error rate. An aggregate chi square statistic was estimated for each set of convergent input parameter values, based on the difference between the predicted and observed values for each calibration target. Probability weights were assigned to set of convergent input parameter values using the reciprocal of the aggregate chi square statistic, to represent the relative accuracy of each set [20].

A Monte Carlo simulation was then undertaken in which 1000 iterations of the model were evaluated. Each iteration involved the random sampling of a convergent set of input parameter values using the defined probability weights to inform the sampling. The 2.5th and 97.5th percentile values for the GP need and supply informed the 95% confidence limits for each scenario.

## Results

**Fig. 2:**
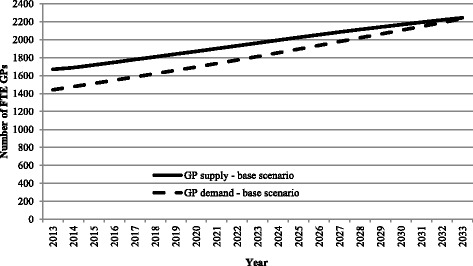
Estimated future supply and demand of FTE GPs in South Australia, 2013 to 2033

The model’s baseline scenario indicates that in aggregate, the GP workforce is currently over supplied by around 230 FTE GPs, but that this over-supply will decline steadily to around 30 by 2033 (Fig. 2). The baseline scenario reflects assumptions that age- and gender-specific disease rates and GP working patterns and exit rates will remain constant. If this baseline scenario is accepted as the best estimate of future need and supply, policy options might focus on how best to speed up the reduction in the over-supply of GPs and redistribute GPs.

### Policy and non-policy scenarios

**Fig. 3:**
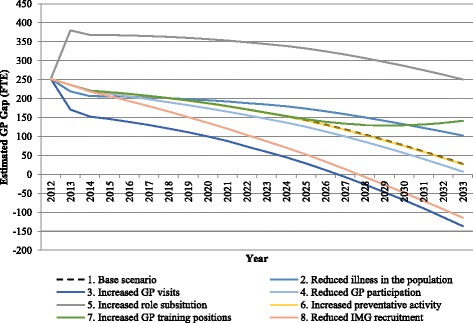
Estimated future supply and demand of FTE GPs in South Australia, 2013 to 2033—single scenarios

The impact of scenarios representing variation in the predicted supply of GPs, need for GP services, and government policies are presented in Table 3 and Fig. 3.

**Table 3:** Summary of FTE GP estimates for South Australia for the single base, policy and non-policy scenarios, 2013, 2023 and 2033

Type of scenario	Scenario		2013	2023	2033
Base	1. Base	Supply	1671	1971	2257
		Demand	1435	1808	2230
		Mean gap (95% CIs)	236 (163, 271)	163 (68, 276)	28 (−93, 260)
Non-policy scenarios	2. Reduced illness in the population	Supply	1671	1971	2257
		Demand	1452	1787	2155
		Mean gap (95% CIs)	219 (158, 256)	184 (81, 305)	102 (−34, 315)
	*3.* Increased GP visits	Supply	1671	1971	2257
		Demand	1501	1912	2394
		Mean gap (95% CIs)	171 (128, 200)	59 (−68, 175)	−136 (−301, 106)
	4. Reduced GP participation	Supply	1671	1955	2237
		Demand	1435	1808	2230
		Mean gap (95% CIs)	236 (206, 268)	147 (37, 255)	7 (−215, 232)
Policy scenarios	5. Increased role substitution	Supply	1671	1971	2257
		Demand	1291	1628	2007
		Mean gap (95% CIs)	380 (340, 414)	344 224, 456)	251 (63, 482)
	6. Increased preventative activity	Supply	1671	1971	2257
		Demand	1436	1809	2231
		Mean gap (95% CIs)	236 (195, 270)	162 (43, 275)	27 (−161, 259)
	7. Increased GP training positions	Supply	1671	1971	2371
		Demand	1435	1808	2230
		Mean gap (95% CIs)	236 (163, 271)	163 (68, 276)	141 (20, 374)
	8. Reduced IMG recruitment	Supply	1671	1896	2115
		Demand	1435	1808	2230
		Mean gap (95% CIs)	236 (163, 271)	87 (−7, 202)	−114 (−236, 114)

The three non-policy scenarios show different outcomes over the projection period (Table 3 and Fig. 3). If age- and gender-specific rates of disease change (reduced illness in the population scenario), the estimates show a smaller surplus of FTE GPs over the next 20 years, although by 2033 this surplus has reduced and moved to a balanced workforce. Similarly, a decrease in participation rate of the male workforce scenario estimates a smaller surplus for the first 20 years, but by 2033 it reaches a balanced workforce. In contrast, the increased GP visits per unit of need scenario estimates a more quickly decreasing surplus, with a shortfall estimated of 136 FTE GPs by 2033 (Table 3 and Fig. 3).

The policy-related scenarios also vary in their results over the projection period (Table 3). Two scenarios estimate a continued surplus over the period—increased role substitution (excess of 251 FTE GPs by 2033) and increased GP training positions (excess of 141 FTE GPs by 2033). Increasing preventative activity by GPs resulted in a move from surplus of 236 FTE GPs to a balanced workforce (27 FTE GPs) while the scenario that reduced the number of IMGs recruited showed that estimates of FTE GP moves from a surplus in 2013 to a shortage in 2033 (Table 3). No single policy option improved on the baseline scenario.

### Combined scenarios

The impact of different policy options and the influence of other non-policy options can be tested when scenarios are combined. This can be illustrated using two of the single non-policy scenarios—increased GP visits and reduced illness in the population. Furthermore, it can be illustrated with the combination of two single non-policy scenarios—increased GP visits and decreased GP participation—and one of the policy scenarios—reduced IMG recruitment.

**Fig. 4:**
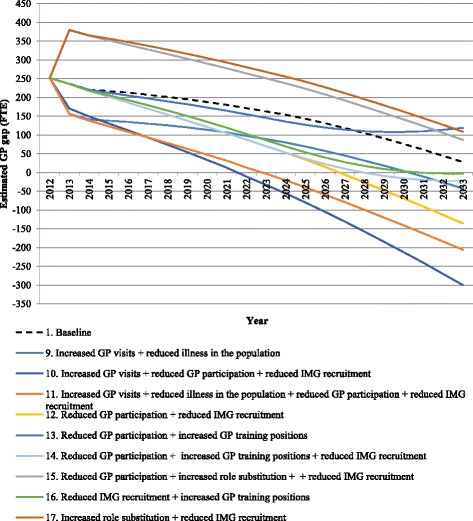
Estimated future supply and demand of FTE GPs in South Australia, 2013 to 2033—combined scenarios

In the increased GP visits scenario based on current projections, the workforce was estimated to a move from a surplus in 2013 through to a balanced workforce in 2023 to a shortfall of 136 GPs in 2033. However, these estimates change if increased GP visits occur with changes in illness in the population, based on an overall reduction illness. The result is a small surplus in 2023 (89 FTE GPs) and a balanced workforce in 2033 (−42 FTE GPs) (Table 4), suggesting that reduced illness will offset the increased demand for GP visits. If increased GP visits are combined with reduced GP participation rates (non-policy scenario) and reduced IMG recruitment (policy scenario), the result is an estimated shortage of 300 FTE GPs in 2033. This is the worst-case scenario and is driven by increased demand and a reduced IMG pool. However, this outcome could be reduced if changes in illness in the population occur with these other scenarios, reducing the shortage to 206 FTE in 2033 (Scenario 3 on Table 4). A graph of the combined scenarios is provided in Fig. 4.

**Table 4:** Summary of FTE GP estimates for South Australia for selected single scenarios when combined with other policy and non-policy scenarios, 2013, 2023 and 2033

Non-policy scenario	Scenario		2013	2023	2033
Increased GP visits	*Increased GP visits* (*single scenario 3*)	*Supply*	*1671*	*1971*	*2257*
		*Demand*	*1501*	*1912*	*2394*
		*Mean gap (95% CIs)*	*171 (128, 200)*	*59 (*−*68, 175)*	−*136 (*−*301, 106)*
	9. Increased GP visits + reduced illness in the population single scenarios 2 + 3)	Supply	1671	1971	2257
		Demand	1515	1882	2300
		Mean gap (95% CIs)	156 (117, 185)	89 (−39, 201)	−42 (−236, 180)
	10. Increased GP visits + reduced GP participation + reduced IMG recruitment (single scenarios 3 + 8)	Supply	1671	1878	2094
		Demand	1501	1912	2394
		Mean gap (95% CIs)	170 (136, 186)	−33 (−155, 76)	−300 (−484, −75)
	11. Increased GP visits + reduced illness in the population + reduced GP participation + reduced IMG recruitment (single scenarios 2 + 3 + 4 + 8)	Supply	1671	1878	2094
		Demand	1515	1882	2300
		Mean gap (95% CIs)	155 (113, 184)	−3 (−116, 104)	−206 (−390, −12)
Reduced GP participation	*Reduced GP participation (single scenario 4)*	*Supply*	*1671*	*1955*	*2237*
		*Demand*	*1435*	*1808*	*2230*
		*Mean gap (95% CIs)*	*236 (206, 268)*	*147 (37, 255)*	*7 (*−*215, 232)*
	12. Reduced GP participation + reduced IMG recruitment (single scenarios 4 + 8)	Supply	1671	1878	2094
		Demand	1435	1808	2230
		Mean gap (95% CIs)	236 (205, 279)	70 (−49, 179)	−136 (−323, 33)
	13. Reduced GP participation + increased GP training positions (single scenarios 4 + 7)	Supply	1671	1954	2348
		Demand	1435	1808	2230
		Mean gap (95% CIs)	236 (190, 262)	145 (31, 253)	119 (−68, 336)
	14. Reduced GP participation + + Increased GP training positions + reduced IMG recruitment (single scenarios 4 + 7 + 8)	Supply	1671	1878	2207
		Demand	1435	1808	2230
		Mean gap (95% CIs)	236 (190, 258)	70 (−42, 177)	−23 (−200, 204)
	15. Reduced GP participation + increased role Substitution + reduced IMG recruitment (single scenarios (4 + 5 + 8)	Supply	1671	1878	2094
		Demand	1291	1628	2007
		Mean gap (95% CIs)	379 (334, 401)	251 (138, 358)	87 (−87, 309)
Policy scenario					
Reduced IMG recruitment	*Reduced IMG recruitment (single scenario 8)*	*Supply*	*1671*	*1896*	*2115*
		*Demand*	*1435*	*1808*	*2230*
		*Mean gap (95% CIs)*	*236 (163, 271)*	*87 (*−*7, 202)*	−*114 (*−*236, 114)*
	16. Reduced IMG recruitment + increased GP training positions (single scenarios 7 + 8)	Supply	1671	1894	2227
		Demand	1435	1808	2230
		Mean gap (95% CIs)	236 (206, 249)	86 (−26, 194)	−3 (−216, 224)
	17. Reduced IMG recruitment + increased role substitution (single scenarios 5 + 8)	Supply	1671	1896	2115
		Demand	1291	1628	2007
		Mean gap (95% CIs)	380 (330, 409)	268 (136, 384)	109 (−108, 347)

Decreased participation rates for the male GP scenario is predicted to move from a surplus to a balanced workforce between 2013 and 2033. When this scenario is combined with policies that reduce the number of IMGs, it is estimated the workforce will move more rapidly to a balanced workforce (i.e. in 2023 rather than 2033), but would result in a shortfall longer term (−136 FTE GPs in 2033) (Table 4, scenario 4). However, these results could be reversed, with a balanced workforce achieved by 2033 if the substitution scenario (policy scenario) is introduced, which assumed 10% of all GP consultations could be provided by practice nurses rather than GPs (Table 3, scenario 7). The best-case outcome where the workforce achieves balance by 2023 and remains balanced to 2033, arose when the GP participation rates scenario was combined with the policy levers of increased GP training positions and reduced IMG recruitment (Table 3, scenario 6). In contrast, if the decreased participation of the workforce occurs with a policy to increase GP training positions, the result is estimated to be a surplus in 2023 and 2033 (Table 3, scenario 4).

### Cost of workforce policies

The estimated costs for the three policy scenarios are shown in Table 5. When compared to the baseline scenarios, the policy of increased training positions was more costly than the baseline scenario, while increased role substitution and reduced IMG recruitment policies resulted in savings (see Table 5). Implementation costs for the policy to increase GP training positions over the 20-year period was AUD$247,296,900 compared to the baseline scenario which was AUD$234,577,101, a difference of AUD$12,719,798. The policy to reduce the number of IMGs recruited saved the government AUD$3,783,291 over the projection period, if no other policy was implemented to offset the reduced number of IMGs (i.e. increased training positions). Using nurses to undertake a small proportion of GP consultations resulted in a government saving of AUD$752,946,586 over the period. The cost was also estimated for two combined scenarios, where the policy to reduce IMGs was offset by increasing GP training places and through role substitution. When compared to the baseline costs, the combined policy cost to the government of reduced IMG recruitment with increased GP training places, was an additional AUD$8,936,508 over the projection period. For the combination of reduced IMG recruitment and roles substitution, this policy combination saved the government AUD$543,321,440.

**Table 5:** Comparison of estimated total cost of policy and baseline scenarios, 2014–2033, $AUD

Scenarios		Total cost of baseline scenario	Total cost of policy scenario	Difference (baseline – scenario)
Single scenarios	Increased role substitution policy (scenario 5)	$7,529,465,856	$6,989,927,706	−$752,946,586
	Increased GP training positions policy (scenario 7)	$234,577,101	$247,296,900	$12,719,798
	Reduced IMG recruitment policy (scenario 8)	$5,044,388	$1,261,097	−$3,783,291
Combined scenario	Increased GP training positions policy + reduced IMG recruitment policy (scenarios 7 + 8)	$239,621,489	$248,557,996	$8,936,508
	Increased role substitution policy + reduced IMG recruitment policy + (scenarios 5 + 8)	$7,534,510,243	$6,991,188,803	−$543,321,440

In terms of implementation, the policy scenarios related to training positions and IMGs both had a time lag of approximately 3 years before trained graduates enter the GP stock. This is a greater time lag than recruitment of IMGs who can enter the stock within 1–2 years. There would also be a time lag associated with the nurse substitution policy in terms of implementation of the policy and potentially training time required for an increased in demand for nurses.

## Discussion

The model’s baseline scenario indicates that the South Australian GP workforce will become more balanced over the next 20 years with only a small excess of GPs by 2033. The growth in GP supply over this period is driven by an increasing proportion of female GPs making up the GP workforce, from 35% of the FTE workforce in 2013 to 45% in 2033. This estimate is based on the assumption that the current ratio of male to female new graduates remains stable into the future, which is supported by the evidence which shows a consistently greater proportion of female medical students graduating from Australian medical schools [27]. This trend is likely to have the greatest impact on the GP workforce, which is seen as an attractive career by females with shorter, part-time training, compared to other specialists and more flexible working hours once in the workforce [28–30].

With the baseline results indicating a small surplus over the projection period, there would be little need for policy interventions. However, exogenous changes in the future could impact the demand and supply of the GP workforce and change the workforce balance in South Australia. The scenario analysis was able to assess the impact of exogenous or non-policy changes and which policy or policies could be used to address the resultant workforce imbalances.

The scenario that resulted in the largest surplus when compared to baseline by 2033 was that involving practice nurses undertaking a proportion of GP care (policy scenario). The worst-case scenario which resulted in a shortage of GPs by 2033 was the combination of increased GP visits (non-policy scenario) with reduced GP participation rates (non-policy scenario) and less reliance on IMGs (policy scenario). However, for both these scenarios, these changes were modest and may not require significant changes in policies.

The reliance on IMGs to provide GP services is highlighted by the sensitivity of the model’s results to changes in the number of IMGs. A reduction in IMGs by a small amount resulted in SA moving from a position of surplus GPs to one of under-supply. This result also illustrates issues around the distribution of the workforce, an ongoing workforce problem in Australia. The IMGs in this model were entering only the rural workforce and while overall the model estimated that SA would have a surplus of GPs, they are not necessarily working in areas where they are needed. While numerous strategies have been implemented over the last 20 years to increase the number of Australian graduates working in rural and remote areas [2], the model suggests in terms of supply that the uneven distribution of the workforce will continue.

The scenario that focused on increased role substitution where a nurse would undertake a small percentage of consultations rather than a GP not surprisingly saw a reduction in demand for GPs. This outcome may not reflect what would occur in the real world. Research indicates that while nurses can provide high quality care and similar health outcomes to that of a GP, the evidence on reducing a GP’s workload is unclear, given the current payment model [31]. Demand for GPs may not decrease as the use of nurses may allow GPs to meet unmet demand or generate demand for services and that nurses create their own demand for services. The development of an aligned model for practice nurses would allow a better understanding of the impact of this scenario as well as the cost associated with implementing such a policy.

This model used prevalence and incidence as measures of health need in the population and it was expected that increasing levels of chronic disease would result in increased demand for services. However, the scenario analysis did not support this. When changes in prevalence and incidence were modelled using forecasts by Vos et al [32] and Goss [33], the result was a decrease in GP demand when compared to the base scenario. This result was driven by predicted decreases in the incidence of most conditions, increases in prevalence in some conditions (mainly chronic conditions) and population changes which saw increased cases in the older age groups but reductions elsewhere.

The increased preventative activity scenario was nearly identical to the baseline scenario, suggesting that this has little impact on demand for services. However, the benefits of greater preventative activity can result in long-term improvements to health and quality of life that may be better reflected in the reduced illness in the population scenario. Moreover, the impact may be seen in other areas of the health care system which are not part of this model.

Our modelling suggests that the consequences of an increasing number of GPs seeking better work-life balances may not be significant in the shorter term [16, 34]. Our scenario, which assumed an increase in the number of male GPs working in urban areas moving to part-time work based on research by Joyce et al. [34, 35], reduced the surplus and resulted in a balanced workforce by 2033. While this was a non-policy scenario, incentives to move GPs to part-time work could be investigated to reduce existing surpluses.

The non-policy scenario of a modest increase of 5% in the annual number of consultations per prevalent/incident case resulted in a shortage of GPs by 2033 and represented the worst single scenario result. This scenario saw a 27% increase in consultations between 2013 and 2023 and a 25% increase between 2023 and 2033. It follows the trend seen in Medicare Australia data over the last 10 years that has seen GP attendances in SA rise by 26% between 2003 and 2013 and predicted rises in the length and number of GP consultations in Australia by 31% between 2006 and 2020 [18].

Our modelling shows that there are a number of policy responses that could be implemented to counter the potential for an imbalanced GP workforce in SA. Policies focusing on training positions and recruitment of IMGs can have a significant impact on the supply of GPs. Moreover, improved health of the population can counteract the demand of more GP services. Policy levers to change work patterns are more difficult. Role substitution has a clear impact on reducing the number of GPs required, and the use of Medicare items has been used in the past to support an expanded GP nurse role.

### Policy cost

Under the assumptions used in the baseline scenarios, SA is estimated to have a surplus of GPs over the next 20 years, although this surplus reduces over time. As a result, the policy focus is not on which policy would address a predicted shortfall, as is often the case in workforce modelling [13, 36–39], but assessing the impact of non-policy changes and if these would result in a need for a policy intervention and which policy options would be most cost effective. Our analysis of the non-policy scenarios—changing illness in the population, increased GP visits and decreasing GP participation rates—showed that only increased GP visits would require a direct policy intervention. Two policy interventions could offset the predicted shortfall—increasing GP training positions or the use of role substitution.

For governments, the decision on the most appropriate policy interventions to offset workforce shortages or surpluses is determined by how the intervention will increase the workforce or slow the growth of the workforce in terms of absolute numbers but also the cost of the intervention. We have attempted for the first time to provide not only an assessment of the policies in terms of impact on FTE GPs, but also the cost of the interventions, allowing more informed decision-making.

Not surprisingly, increasing the number of GP training positions is the most costly policy, particularly as in our modelling the additional places were introduced in 2015, resulting in an additional 183 GP training positions over the 18 years.

The most cost-effective policy intervention is increasing role substitution. Expanding the role of practice nurses to undertake an increased number of consultations is a less expensive policy than training additional GPs. However, the implementation of such a policy assumes that there are sufficient numbers of trained practice nurses who can accommodate an increase in workload and this may not be the case. Therefore, a more complex cost analysis should include the cost of nurse training. Moreover, while nurses may take on consultations within their scope of practice, the GP consultation patterns may also change, which could lead to more costly GP attendances.

Predictably our cost analysis of the reduced IMG recruitment policy indicated savings to the government. The reduction in the scope of a current policy will understandably reduce the cost to the government. However, this is too simplistic as it assumes that there is an alternative workforce to fill the gap left by IMGs or that the demand has reduced. This may require training additional GPs which then adds to the cost of this policy. Our analysis indicates that if the reduced IMG recruitment policy is implemented in conjunction with increased GP training positions to cover the gap, the policy will become more costly. The cost of recruiting an IMG is much smaller than the cost of additional training places, and more importantly, the practice location of the IMGs can be controlled through a legislative process such as the use of areas of workforce need. In contrast, we know that while we have increased the number of GP training positions, the recruitment of Australian graduates to work in rural and remote areas remains difficult [2].

While the Australian government has focused on recruiting Australian graduates to areas of workforce need [40], a policy of expanding role substitution may be worthy of consideration for these areas. Our cost analysis indicates that using nurses to fill the workload left by reducing IMGs will provide considerable savings in terms of policy costs. We know that rural and remote nurses already take on a broader range of services due to the lack of GPs and so expanding this further may be worth consideration [41]. However as discussed above, this assumes that there are sufficient numbers of nurses available and they are appropriately trained to take on these additional roles. The use of advance nurses or nurse practitioners is seen as an alternative workforce to doctors in rural and remote areas [42]. At the same time, there is a lack of sufficient evidence at the moment for policy recommendations for role substitutions [43].

### Limitations

The main limitation is common to all modelling exercises, that the projections are based on parameter estimates informed by data sources of varying quality and relevance. While Australia has collected data on the medical workforce over a long period, this is cross-sectional data and does not allow for tracking of GP cohorts over time. Similarly, the projected changes in disease prevalence and incidence were based on data from 2003, and did not capture all illnesses and injuries which are likely to underestimate need for GP services. Another example is the 10-year-old data describing the proportion of male and female GPs working part-time. We know that working hours are changing but we do not know how these may change further in the next 20 years.

The analysis tested the effects of a range of policy and non-policy influenced scenarios, but these were not an exhaustive set of possible events and actions that could influence demand for and supply of health services into the future. A wide range of new models of care or health care technologies may arise that will impact on the reported predictions, but we chose to test scenarios for which some evidence was available to inform expected impacts on demand and supply.

While the model accounted for urban and rural location in the supply sub-model, this was not incorporated in the need sub-model and so the model did not assess imbalances in the distribution of GPs across the state of SA. Issues in attracting GPs to practice in rural areas is a longstanding issue across Australia and so despite predictions of an over-supply of GPs in aggregate, it is likely that the baseline scenario actually reflects increasing under capacity in rural areas. This is an area for further development, and future versions of the model will incorporate both need and supply in urban and rural areas.

The costing analysis had a number of limitations. In many cases it was based on published expenditure data for policies and may not have accounted for all costs associated with a policy. For example, the cost of recruiting an IMG used program funding data which may not have included costs such as additional training that may be required for an IMG. In estimating the costs of role substitution, we used an average cost based on existing MBS nurse items. This assumes that such a cost would apply to all their consultations with no variation that reflected the complexity of different consultations and so differing lengths of consultations. We also did not include in our calculation the implementation cost associated with introducing nurse substitution or the cost of training additional nurses. The analysis also did not consider the effect of retention rates on the cost of the policies. For example, while IMGs may be less expensive to recruit, if they remain for a shorter period in rural areas compared to Australian graduates, the long-term cost of the policy is likely to be greater. A more detailed cost analysis of all the policies warrants further research.

## Conclusions

Over the next 20 years, our baseline scenario predicts that South Australia’s GP workforce will move towards equilibrium, with need and supply of GP services being well balanced. However, further consideration of geographical variations in the balance of need and supply within and between urban and rural areas is required. However, alternative scenarios result in less balanced projections. Additionally, the cost of policy interventions varied. In the absence of better quality data, expert consideration of the relative likelihood of alternative non-policy scenarios and the broader impact of policy options may best inform appropriate policy responses. In addition, the workforce model presented in this paper should be updated at regular intervals to inform the need for policy intervention.

## Additional file

Additional file 1: Graphic representation projected population for South Australia. This includes two figures. Figure S1: Projected population for South Australia (Series B), females by age groups in South Australia, 2013-2033. Figure S2: Projected population for South Australia (Series B), males by age groups in South Australia, 2013-2033. (DOCX 21 kb)
