# Supplementary material for: What will make a difference? Assessing the impact of policy and non-policy scenarios on estimations of the future GP workforce
Source: Hum Resour Health. 2017 Jun 28;15:43. doi: 10.1186/s12960-017-0216-1 (PMC5490216; doi:10.1186/s12960-017-0216-1)
Supplement: Additional file 1: — Graphic representation projected population for South Australia. This includes two figures. Figure S1: Projected population for South Australia (Series B), females by age groups in South Australia, 2013-2033. Figure S2: Projected population for South Australia (Series B), males by age groups in South Australia, 2013-2033. (DOCX 21 kb) [file 12960_2017_216_MOESM1_ESM.docx]

Additional File 1: Graphic representation projected population for South Australia

Figure 1: Projected population for South Australia (Series B), females by age groups in South Australia, 2013-2033

Figure 2: Projected population for South Australia (Series B), males by age groups in South Australia, 2013-2033
